# Ischiorectal Approach to Cryoablation of the Pudendal Nerve Using a Handheld Device: A Report of Two Cases

**DOI:** 10.7759/cureus.44377

**Published:** 2023-08-30

**Authors:** Hunter Hampton, Arun Kalava

**Affiliations:** 1 Anesthesiology, University of Central Florida College of Medicine, Orlando, USA

**Keywords:** chronic pain management, interventional pain medicine, pudendal nerve, percutaneous cryoablation, pudendal neuralgia

## Abstract

The pudendal nerve is situated deep within the pelvis and is a challenge to target for pain interventions due to the theoretical risk of incontinence with manipulation. The management of pudendal neuralgia using cryoablation is currently limited as it has historically required computed tomography (CT) guidance by interventional radiologists. Through this report, we describe a safe, reproducible, ischiorectal fossa approach to pudendal nerve cryoablation with a handheld device utilizing anatomical landmarks, nerve stimulation, and fluoroscopy. Two patients with longstanding pelvic pain and positive response to diagnostic pudendal nerve blocks underwent bilateral cryoablation of the pudendal nerves. This procedure was performed with the patients in prone positioning and percutaneous insertion of a cryoablation probe medial to the ischial tuberosity and lateral to the rectum. Correct positioning on the pudendal nerve was achieved with nerve stimulation eliciting visible anal sphincter contraction, and fluoroscopic imaging of the probe relative to the ischial spine. The probe was set to -88 Celsius for 108 seconds and a total of two cycles were performed. Pain reduction was reported for 3-4 months and repeat cryoablation was similarly efficacious with no evidence of incontinence. This technique, we believe minimizes risks and simplifies cryoablation to be performed on an outpatient basis by more pain physicians.

## Introduction

Pelvic pain can be multifaceted in origin. The pudendal nerve is unique in that it innervates many regions of the pelvis such as the genitalia, anus, and buttocks. Patients who have had no response to conventional management of their pelvic pain may benefit from targeted procedures aimed at the pudendal nerve. Pain physicians are limited in the procedures they can offer patients with pudendal neuralgia. Sacral neuromodulation by implantable nerve stimulator or large-scale decompressive surgery are possible interventions indicated after conservative treatments fail [1]. Cryoablation has distinct advantages by providing sustained pain relief in a less invasive manner but traditionally has required computed tomography (CT) guidance [2].

This report aims to demonstrate the feasibility and safety of pudendal nerve cryoablation through a combined anatomic landmark, fluoroscopy, and nerve stimulator-guided approach. Two patients provided written consent for inclusion in this report highlighting their extensive history of chronic pelvic pain, failed conservative pain reduction strategies, and positive response to pudendal nerve blocks. They subsequently underwent a minimally invasive cryoablation of bilateral pudendal nerves with a handheld device, using the ischiorectal approach. These patients attained significant pain reduction and reported no anal sphincter dysfunction.

Indications

A diagnostic pudendal nerve block is useful in determining which nerves are associated with pelvic pain transmission and the potential efficacy of nerve targeting procedures. Transvaginal pudendal nerve blocks are commonly performed in females as a result of the easily identifiable anatomic landmark of the ischial spine. In men, access to the pudendal nerve is comparatively difficult and requires a modified approach to which physicians may be unfamiliar. The perirectal approach involves palpating the ischial spine with the index finger in the rectum and inserting a needle lateral to the rectum and advancing to the depth of the ischial spine as confirmed via fluoroscopy or nerve stimulator-induced contraction of ipsilateral external anal sphincter [3]. An ischiorectal approach to pudendal nerve block is also possible by external palpation of the ischial tuberosity with nerve stimulator positional confirmation.

The reduction or resolution of pain post-procedurally is indicative of pudendal nerve involvement in the patient’s pain transmission and implicates pudendal neuralgia [4]. Following this confirmatory procedure, options like nerve stimulator implantation [5] or surgical decompression, neuro-ablation by pulsed radiofrequency [6] or cryoablation [2] have been described. Both percutaneous cryoablation and pulsed radiofrequency ablation (pRFA) have been evidenced to be highly efficacious in the treatment of chronic pudendal neuralgia [1]. pRFA shares similarities to cryoablation through its selectivity of small diameter A-delta and C nociceptive fibers while sparing motor fibers [7]. Conversely, continuous radiofrequency ablation poses an increased risk of damage to motor fibers as a result of sustained elevated tissue temperatures greater than 42 °Celsius and is thus a poor modality for analgesia of mixed nerves [7].

Cryoablation is the application of extremely low temperatures that reversibly ablates neural tissue to the point of decreased sensory transmission while retaining motor function [8]. In contrast to heat ablation, there is a decreased risk of neuroma formation due to the lack of disruption of both the perineurium and epineurium which allows for nerve regeneration [9]. Cryoablation provides prolonged periods of pain reduction [2] and can be repeated as a result of this nerve regenerative effect. Previously, this was only possible following surgical exposure of the desired nerve [10]. However, handheld cryoablative devices are now available that allow for percutaneous probe access [11]. This enables procedures to take place on an outpatient basis. The procedural algorithm for managing pelvic pain should include cryoablation, considering its efficacy and ease of deployment. However, the pudendal nerve is situated deep within the pelvis which has long made such procedures unfeasible to physicians without CT guidance.

Previous descriptions of pudendal nerve cryoablation have noted the need for CT guidance by interventional radiologists, citing precision advantages with real-time 3-dimensional guidance [12]. The risks of anatomical or fluoroscopically guided percutaneous access have been unnecessarily skewed by the perception that limited probe visibility decreases safety without the ability to monitor the ablation zone in real time [10]. While CT imaging does yield high cross-sectional precision, both modalities have similar safety profiles.

Procedural anatomy

The pudendal nerve originates from the ventral spinal nerve roots S2-S4 and travels alongside the sciatic nerve as it traverses through the greater sciatic foramen. It diverges from the sciatic nerve across the sacrospinous ligament and ischial spine posteriorly before continuing through the lesser sciatic foramen. The pudendal nerve now divides into the first of its three branches, the inferior rectal nerve, and subsequently into the perineal nerve and dorsal nerve of the penis/clitoris. The relevant vasculature of this region involves the internal pudendal artery and vein which course alongside the pudendal nerve within the Alcock canal, the fascial tunnel that courses posteroinferior to the sacrotuberous ligament [13]. Further structures to note include the inferior gluteal artery and vein which track laterally to the ischial spine and tuberosity, and the posterior femoral cutaneous nerve which passes more superficial and lateral to the sacrotuberous ligament [14]. The majority of ischiorectal fossa comprises fat. It also houses the pudendal nerve and internal pudendal vessels, along with the perineal branch of S4, the perforating cutaneous nerve, and lymphatic trunks [15]. 

Percutaneous probe insertion posteromedial of the ischial tuberosity in the ischiorectal fossa for pudendal nerve access is thus anatomically advantageous due to a lack of significant structures that risk injury. The ischial tuberosity is sufficient by palpation alone for appropriate inferomedial insertion. Body habitus may complicate the palpation of the ischial tuberosity and can be assisted by ultrasound to locate this bony landmark. Upon insertion, ultrasound and fluoroscopy are useful in confirming positionality in relation to the ischial spine and with such anatomical and imaging modalities, there is reasonable assurance that probe placement is accurate to the region of the Alcock canal.

Cryoablation, however, requires more than just regional accuracy to ensure effective analgesia [16]. The area of effect for the Iovera (Pacira Biosciences Inc, Parsippany, NJ) device is a 1 cm circumferential ice ball and, therefore, requires precise probe placement within such a range. Additionally, there is the risk of unwanted cryoablation of the sciatic nerve located anterolateral to the Alcock canal at the level of the greater sciatic foramen. This necessitates exact probe positioning on the pudendal nerve without sciatic nerve involvement. Fluoroscopic imaging can be used as an effective alternative to CT guidance that provides accurate localization to the pudendal nerve [17]. The use of a nerve stimulator is also an effective means of targeting the pudendal nerve accurately to safeguard improper positioning [18]. The pudendal nerve is a mixed nerve that provides both sensory and motor innervation to the anal sphincter. So, upon nerve stimulation with probe placement on the pudendal nerve, the anal sphincter will contract. Accordingly, nerve stimulation with incorrect positioning on the sciatic nerve will yield contractions of lower extremity musculature innervated by the sciatic nerve. These concurrent functional and imaging cues provide confirmatory positional input to facilitate probe repositioning in order to begin cryoablation.

## Case presentation

Patient A: burning micturition and distal penile pain

A 53-year-old male with a past medical history of hypercholesterolemia and colon polyps presented with long-standing penile pain along with burning micturition for 10 years. The pain was, burning, located at the distal end of the penis and did not radiate along the shaft. It was 6-8/10 on the numerical rating scale (NRS). There was no reported prior trauma and no lesions or erythema present on the penis. The pain was worsened with defecation and relieved to some extent with amitriptyline 50mg once daily. Pelvic physical therapy for many months provided minor relief and the relief was non-sustained by continued therapy. The patient had been in a 25-year-long monogamous relationship and had tested negative for sexually transmitted infections. Previous workup included mildly elevated prostate-specific antigen that led to prostate biopsies that were found to be negative and subsequent bladder biopsies were similarly negative. Pelvic and genital exams were unremarkable. A rectal exam was deferred.

The patient underwent bilateral pudendal nerve blocks with 4 ml of 1% lidocaine and 10 mg of dexamethasone being administered on each side. The nerve block was performed using the anatomical, landmark and nerve stimulator-guided approach similar to the cryoablation procedure. This resulted in a 60% reduction in burning micturition pain and a 75% reduction in distal penile pain for a duration of 1 week. This was considered a positive diagnostic indicator of pudendal neuralgia. The patient was provided with options of repeat nerve blocks, peripheral nerve stimulation, or cryoablation. The patient opted for cryoablation after a discussion of the benefits and risks associated with the procedure.

Cryoablation was performed on bilateral pudendal nerves at an outpatient surgical center. On follow-up three weeks post operation, the patient reported 85% relief of burning micturition pain and 25% relief of distal penile pain. He was satisfied with the procedure in achieving pain reduction with the only reported side effect being minor inferior scrotal discomfort which shortly resolved and reported no anal sphincter dysfunction. The patient did not return to the clinic until three months later and reported significant pain reduction during this time. He returned for a follow-up nearly four months after cryoablation as the pain began to return and with new areas of perineal pain now present. This prompted a repeat bilateral pudendal nerve cryoablation with similar efficacy of 80% pain relief and no reported anal sphincter dysfunction.

Patient B: vulvodynia and radiating groin pain

A 47-year-old female with a past medical history of fibromyalgia, polycystic ovarian syndrome, T11-12 spinal fusion, and two rectocele repairs presented with constant vaginal & vulvar pain for the past decade after original rectocele repair nine years prior. The vulvar pain was sharp and burning and radiated bilaterally along the groin. The pain was minorly improved with the use of peri-vaginal coconut oil which aided the patient’s ability to sleep. A vaginal exam was deferred as she had recently been seen by a gynecologist, who ruled out any pathology. Diagnostic bilateral pudendal nerve blocks were performed with 4 ml of 1% lidocaine, which resulted in a significant >50% reduction in pain for several days. Corticosteroid was not used as she had allergies to steroids. The positive finding of pudendal neuralgia was conveyed to the patient and a discussion ensued of the previously described therapeutics and their benefits and risks which resulted in the patient opting for bilateral pudendal nerve cryoablation.

After undergoing bilateral pudendal nerve cryoablation, the patient presented at a three-week post-operative follow-up appointment, having received no pain reduction. However, at the two-month post-operative appointment, the patient reported a >40% relief of deep vaginal pain, four weeks after cryoablation, with lingering vulvodynia and radiating groin pain that had not abated. The radiating groin pain initially responded to ultrasound-guided bilateral ilio-inguinal nerve block for a few days and a subsequent thermal ablation at 80 °Celsius for two minutes resulted in near complete resolution of groin pain for many months. The patient returned four months after cryoablation as her deep vaginal pain started returning. She underwent another session of bilateral pudendal nerve cryoablation resulting in >50% pain relief with no reported anal sphincter dysfunction. At the time of this article write-up, both patients seemed to be doing well, with good control of their pelvic pain.

Procedural description

**Figure 1:**
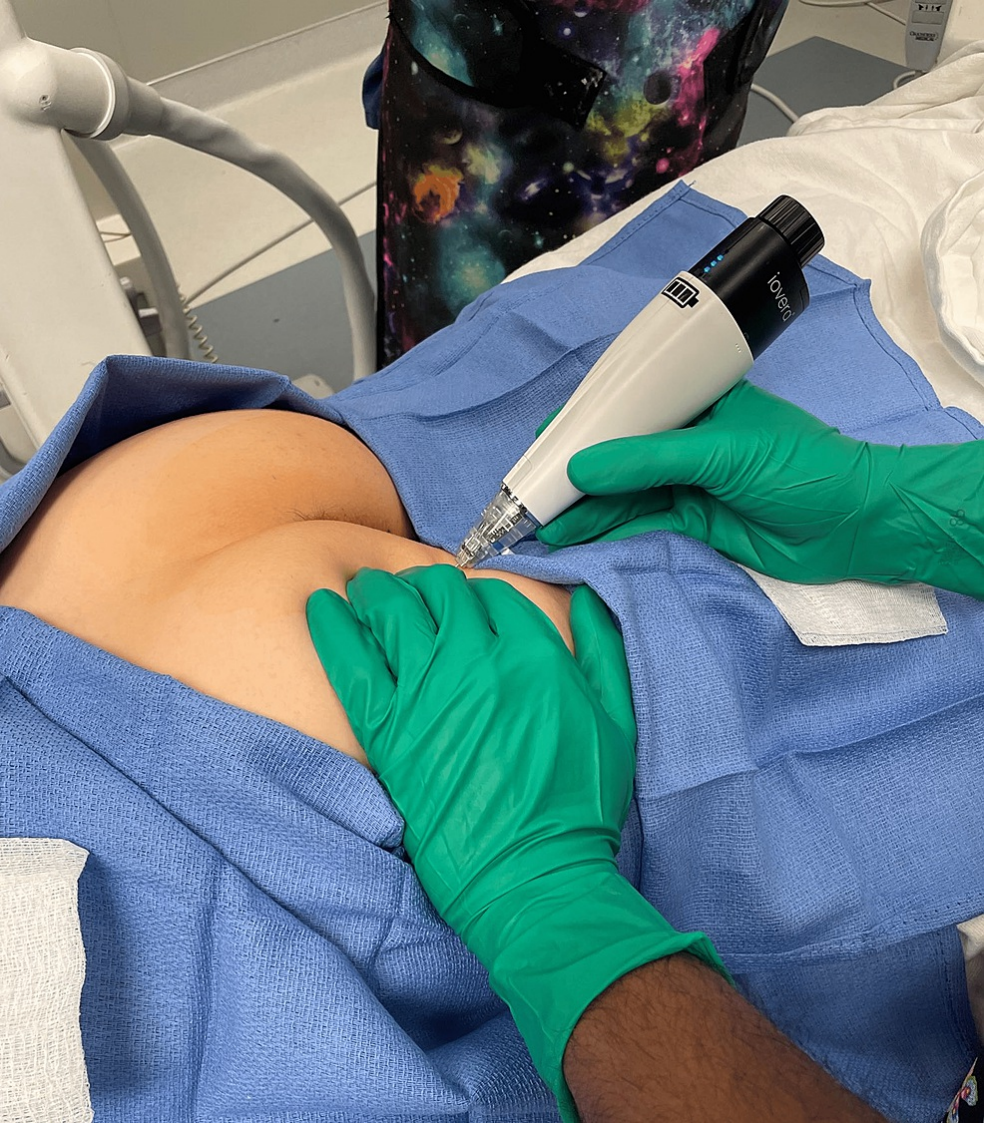
Ischiorectal approach for pudendal cryoablation Used with permission by the American Society for Post-Surgical Pain (ASPSP)

**Figure 2:**
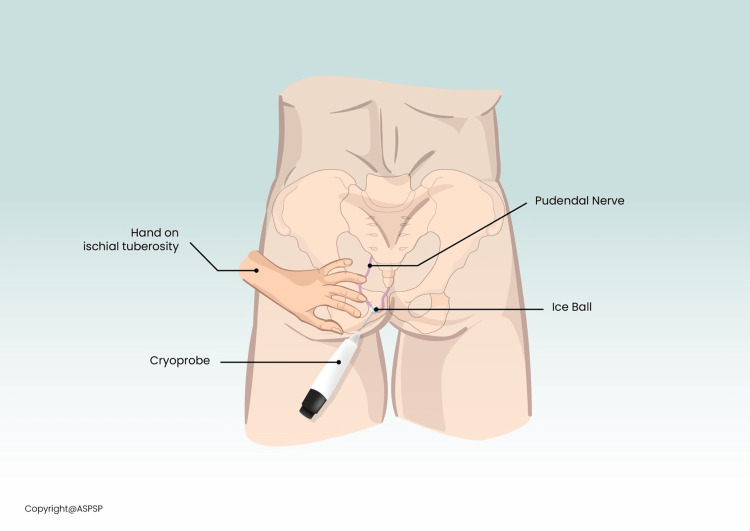
Anatomic illustration of the ischiorectal approach to pudendal nerve cryoablation Used with permission by the American Society for Post-Surgical Pain (ASPSP)

**Figure 3:**
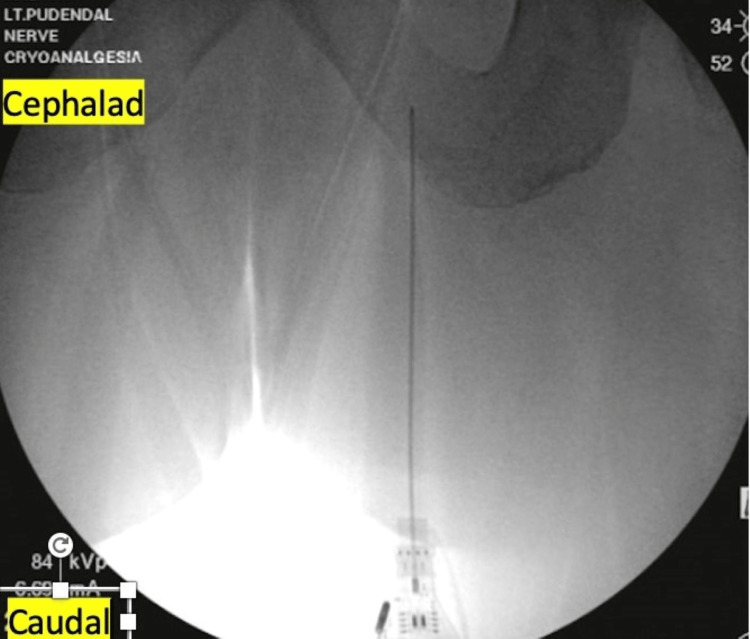
Fluoroscopic image The image shows a pudendal cryoablation needle medial to the right ischial tuberosity in the ischiorectal fossa. Used with permission by the American Society for Post-Surgical Pain (ASPSP)

After obtaining written informed consent, the patient was placed in a prone position at an outpatient surgical center operating room and was sedated with intravenous propofol without paralytics or intubation. The buttocks were exposed, prepared, and draped in an aseptic fashion. The non-operating hand of the physician was used to palpate the ischial tuberosity, pushing away the skin and soft tissue laterally. Inferomedial to the non-operating hand, i.e., between the ischium and the anus, the skin was anesthetized with 1% lidocaine using a 27-gauge hypodermic needle. Then an Iovera (Pacira Biosciences Inc, Parsippany, NJ) cryoablation needle, attached to a nerve stimulator (Pajunk, Geisingen, Germany) was inserted parallel to the plane of the buttock for about 3-6 cm, advancing slowly until a visible anorectal twitch was appreciated at 2-3 mAmp and 1 Hz frequency (Figures 1, 2). Fluoroscopic imaging was also used to gauge the needle depth and its relation to the ischial spine (Figure 3). The C-arm of the fluoroscope was oriented anterior-posterior with no cephalad, caudal, or oblique tilt. The needle was repositioned with structural cues from repeat fluoroscopic imaging and functional cues as provided by confirmatory anorectal twitch. The nerve stimulator output was reduced to 0.5mA and a sustained anal twitch indicated correct positioning on the pudendal nerve. The lesion was then carried out at -88 °Celsius for 108 seconds and a total of two cycles were performed. Disappearance of the anorectal twitch was noted a few seconds into the cryoablation, indicating disruption of neural signals.

## Discussion

Cryoablation presents itself as a pain intervention with unique advantages. The benefits showcased in this report were sustained and repeatable reductions of pain through a convenient outpatient procedure. The patients reported minimal pain lasting approximately four months, with repeat cryoablation yielding similar results as the initial procedures. This method is superior to repeat nerve blocks given the increased frequency required for nerve blocks to achieve consistent analgesia [3]. Sacral neuromodulation and surgical interventions are often less desirable to patients hoping to avoid more invasive procedures. The sustained analgesic efficacy and convenience clearly favor cryoablation when comparing modalities and lead patients to opt for cryoablation over the currently available pudendal neuralgia therapies.

What prevents large-scale adoption of cryoablation in the treatment of pudendal neuralgia is the perceived difficulty of nerve access. While CT imaging can be utilized to ascertain probe positioning in real-time, this is outside the scope of practice of a conventionally trained pain physician. The showcased technique is simple, such that any pain proceduralist would be able to perform pudendal cryoablation in a minimally invasive fashion without the need for CT guidance. The ischial tuberosity and anus are sufficient landmarks to direct initial percutaneous probe insertion, and fluoroscopy is a practical substitute for CT guidance that can determine the proper depth to the level of the ischial spine and pudendal nerve [17]. Furthermore, fluoroscopy minimizes unnecessary radiation exposure by providing a lower dose of radiation compared to CT imaging. These practices replace the need for cross-sectional imaging and allow pain physicians a simple means of access to the pudendal nerve with less radiation exposure.

The associated risks of any percutaneous procedure include bleeding risk and infection with the addition of incontinence and sciatic nerve injury, given this specific procedure. These complications are rare and have not been reported in previous iterations of percutaneous pudendal nerve cryoablation [2]. Similarly, such complications were not observed in these two cases. Continence was preserved even in the setting of direct probe placement on the pudendal nerve as confirmed by anal twitch. This further supports the safety of cryoablation on mixed nerves to selectively ablate sensory fibers while sparing motor fiber function [8]. The ischiorectal approach proved to be ideal in avoiding structural injury to the large vessels, like the inferior gluteal artery, by percutaneous insertion in a plane devoid of interfering vasculature. Nerve stimulation provided an adequate safeguard against sciatic nerve injury as stimulation demonstrated only external anal sphincter contraction without lower extremity involvement.

The two patients included in this report highlight the spectrum of results and intricacies involved in the treatment of chronic pain transmission. Patient A achieved major pain reduction for significantly longer as compared with the previous diagnostic nerve block. Upon pain recurrence, these effects were once more sought after with a repeat cryoablation. Patient B experienced delayed onset pain reduction that did not entirely address her chronic pain symptoms. This experience contrasts the previous reports of pudendal cryoablation that demonstrate analgesia as soon as 24 hours after pudendal cryoablation and up to six months after such a procedure [2]. After subsequent diagnostic nerve blocks of the bilateral ilioinguinal nerves were found to be positive, it was apparent that her sensation of pain involved a multineural pathway. Regardless, aspects of her pelvic pain did respond well to pudendal cryoablation such that she also underwent repeat cryoablation.

## Conclusions

Cryoablation seems to be efficacious in the treatment of pudendal neuralgia by providing persistent and repeatable pain relief. The ischiorectal approach to pudendal nerve cryoablation offers pain interventionalists a simplified procedure to accurately target the pudendal nerve without increased risks of incontinence or unintentional injury to nearby structures. The use of anatomic landmarks, fluoroscopy, and nerve stimulation allows for access to a deep nerve once considered unapproachable without open surgery or CT imaging.
